# Deletion of *Porcn* in Mice Leads to Multiple Developmental Defects and Models Human Focal Dermal Hypoplasia (Goltz Syndrome)

**DOI:** 10.1371/journal.pone.0032331

**Published:** 2012-03-06

**Authors:** Wei Liu, Timothy M. Shaver, Alfred Balasa, M. Cecilia Ljungberg, Xiaoling Wang, Shu Wen, Hoang Nguyen, Ignatia B. Van den Veyver

**Affiliations:** 1 Department of Obstetrics and Gynecology, Baylor College of Medicine, Houston, Texas, United States of America; 2 Molecular and Cellular Biology, Baylor College of Medicine, Houston, Texas, United States of America; 3 Pediatrics, USDA/ARS Children's Nutrition Research Center, Houston, Texas, United States of America; 4 Pediatrics-Neurology, Baylor College of Medicine, Houston, Texas, United States of America; 5 Stem Cells and Regenerative Medicine Center, Baylor College of Medicine, Houston, Texas, United States of America; 6 Molecular and Human Genetics, Baylor College of Medicine, Houston, Texas, United States of America; 7 The Jan and Dan Duncan Neurological Research Institute, Texas Children's Hospital, Houston, Texas, United States of America; National Cancer Institute, United States of America

## Abstract

**Background:**

Focal Dermal Hypoplasia (FDH) is a genetic disorder characterized by developmental defects in skin, skeleton and ectodermal appendages. FDH is caused by dominant loss-of-function mutations in X-linked *PORCN*. *PORCN* orthologues in Drosophila and mice encode endoplasmic reticulum proteins required for secretion and function of Wnt proteins. Wnt proteins play important roles in embryo development, tissue homeostasis and stem cell maintenance. Since features of FDH overlap with those seen in mouse Wnt pathway mutants, FDH likely results from defective Wnt signaling but molecular mechanisms by which inactivation of *PORCN* affects Wnt signaling and manifestations of FDH remain to be elucidated.

**Results:**

We introduced intronic *loxP* sites and a *neomycin* gene in the mouse *Porcn* locus for conditional inactivation. *Porcn-ex3-7flox* mice have no apparent developmental defects, but chimeric mice retaining the neomycin gene (*Porcn-ex3-7Neo-flox*) have limb, skin, and urogenital abnormalities. Conditional *Porcn* inactivation by *EIIa*-driven or *Hprt*-driven Cre recombinase results in increased early embryonic lethality. Mesenchyme-specific *Prx-Cre*-driven inactivation of *Porcn* produces FDH-like limb defects, while ectodermal *Krt14-Cre*-driven inactivation produces thin skin, alopecia, and abnormal dentition. Furthermore, cell-based assays confirm that human PORCN mutations reduce WNT3A secretion.

**Conclusions:**

These data indicate that *Porcn* inactivation in the mouse produces a model for human FDH and that phenotypic features result from defective WNT signaling in ectodermal- and mesenchymal-derived structures.

## Introduction

Focal dermal hypoplasia (FDH), also known as Goltz syndrome or Goltz-Gorlin syndrome, is an X-linked disorder that predominantly affects females. Individuals with FDH have a pleiotropic phenotype consisting primarily of defects of skeleton, skin, and ectodermal appendages. They often have ectrodactyly, syndactyly, brachydactyly, and oligodactyly of hands and feet, and can have shortened or absent long bones combined with typical streaks of atrophic, hypo-, and hyperpigmented skin with abnormal subcutaneous fat deposition in a thin dermis. Other common features consist of eye and ear abnormalities, brittle and sparse hair, dystrophic nails, hypodontia, and supernumerary nipples. More variable findings in FDH include short stature, pointed chin, cleft lip and palate, osteopathia striata of long bones, diastasis pubis, kidney abnormalities, abdominal wall defects, and papillomas of lips, gingivae, and tonsils. Strikingly, central nervous system abnormalities and intellectual disability are uncommon in FDH [1], [2], [3], [4], [5], [6]. It has been suggested that the phenotype of FDH results from a developmental defect in signaling between ectoderm and mesoderm [7].

We and others first described that FDH is caused by mutations in the X-linked *PORCN* gene, which has facilitated the clinical diagnosis of FDH [8], [9], [10], [11], [12], [13]. Females with FDH have heterozygous or mosaic loss-of-function mutations or large deletions of *PORCN* and the few affected males have mosaic mutations [14]. This explains the female predominance of the disorder, absence of male-to-male transmission, and presumed male lethality of germline mutations [1], [15]. *PORCN* is the human orthologue of the *Drosophila* segment polarity gene, *porcupine* [16]. The Porcupine protein is a multipass transmembrane endoplasmic reticulum (ER) protein and a member of the superfamily of membrane-bound O-acyl transferases (MBOAT) [17]. Drosophila and mouse Porcupine lipid-modify Wnt proteins by covalently linking palmitoleic acid at a conserved serine residue (S209 in mouse Wnt3a), which is required for the attachment of a second acyl group, palmitate at a conserved cysteine (C77 in Wnt3a) [18], [19], [20]. This is important for processing, secretion, and signaling of Wingless (Wg) in Drosophila, Wnt3a in mouse, and likely several other Wnt proteins (Wnt1, 3, 4, 5a, 6, and 7b) that have wide temporo-spatial expression and signal via the canonical Wnt/β-catenin and the non-canonical planar cell polarity pathway [18], [19], [20], [21], [22], [23], [24], [25].

*Porcn* has an essential role in early embryonic development [26]. It is first expressed in epiblast cells of peri-gastrulation-stage embryos and later becomes more restricted dorsally, laterally, and in the primitive streak region, with reduced expression in the anterior visceral endoderm. At later stages, expression is stronger dorsally, in the neural tube, cranial region, and optic vesicles and largely overlaps expression patterns of Wnt proteins [27]. It has also been demonstrated in studies on embryos formed by aggregating *Porcn*-mutant embryonic stem (ES) cells with tetraploid blastocysts as well as *in vitro* in cultured cells that the gene is essential for gastrulation and that its inactivation causes failure of endoderm and mesoderm differentiation [25], [27].

To better understand the molecular mechanisms that cause the multiple defects of FDH and to study the function of *Porcn* in an *in viv*o mammalian model, we generated a conditional allele of *Porcn* in mouse ES cells to produce a tissue-specific deletion of the gene. We report here that hemizygous constitutive inactivation of *Porcn* causes early embryonic lethality of male embryos, while heterozygous female embryos have developmental defects of the neural tube and body wall, with most not surviving to birth. Ectodermal-specific inactivation in the skin causes alopecia, thin skin, and tooth anomalies. Inactivation in mesenchyme of the limbs causes shortening of long bones and digits. Cell-based assays demonstrate reduced secretion of WNT3A in the presence of mutant *PORCN*. These combined findings support that defective Wnt signaling is at the basis of the phenotypic features of FDH.

## Results

### FDH-like developmental defects in a chimeric *Porcn* genetrap mutant mouse

**Figure 1:**
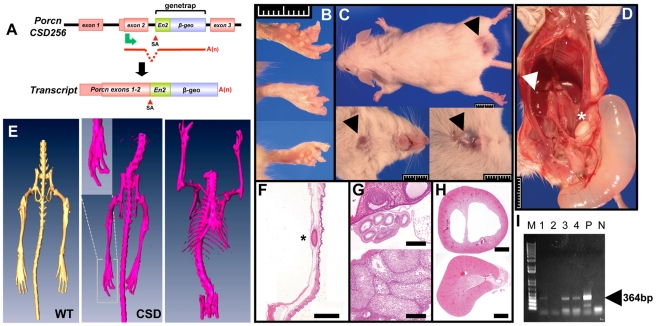
Phenotype of chimeric *Porcn* genetrap mouse. (**A**) *Porcn* genetrap construct CSD256. Top: A genetrap cassette with a strong splice acceptor (SA) upstream of an engrailed2 leader sequence (*En2*) fused to a *β-galactosidase-neomycin* (β-*geo*) gene is inserted in intron 2 of *Porcn*. Bottom: resulting genetrapped fusion transcript that truncates *Porcn* after exon 2 (green arrow, translation initiation site, A(n) poly-A tail). (**B**) Fused and hypoplastic shortened digits. (**C**) Areas of hypoplastic skin with alopecia (black arrows) and swelling at lower back. (**D**) Intra-abdominal cystic structure (likely dilated vas deferens), right hydronephrosis (arrow), testicle and seminiferous tubules (*). (**E**) Skeletal microCT images showing fusion and hypoplasia of digits in mutant mouse (WT, wild type mouse; CSD, mouse generated from CSD256; inset, enlargement of hindpaw). (**F**) Hypoplastic skin with dermoid cyst (*). Scale bar represents 1 mm. (**G**) Top panel: ovary and oviducts (scale bar represents 200 µm). Bottom panel: hypoplastic testis (scale bar represents 100 µm). (**H**) Top panel: hydronephrotic kidney with thin medulla. Bottom panel: contralateral normal kidney (scale bars represent 1 mm). (**I**) Gel picture showing amplification of lacZ (364 bp fragment) in various tissues. M, 1 kb ladder; 1, skin with lesion; 2, normal skin; 3, liver; 4, tail; P, positive control (CSD256 ES cell DNA); N, negative control (C57BL6 gDNA).

We performed two sets of injections into blastocysts of mouse ES cells (line CSD256) that were targeted with a genetrap cassette containing a splice-acceptor sequence upstream of a *β-galactosidase-neomycin* fusion (*β-geo*) reporter gene inserted 3′ to exon 2, which contains the translation initiation site of *Porcn* (**Figure 1A**). Consistent with the prediction that this allele would result in inactivation of *Porcn*, only a single liveborn male chimera (**Figure 1B–I**) was found in 28 offspring. This animal had fused and hypoplastic digits on all 4 extremities (**Figure 1B and 1E**), 2 midline ventral wall defects, and a lower dorsal skin defect with alopecia (**Figure 1C**). The skin lesions showed a thin epidermis with presence of a dermoid cyst in one of the ventral lesions (**Figure 1F**). Interestingly, this mouse also had external male genitalia, but was a hermaphrodite with a single ovary with oviduct and a hypoplastic testis (**Figure 1G**). It developed a cystic intra-abdominal structure, suspected to be a dilated obstructed vas deferens (**Figure 1D**), and had a hydronephrotic kidney (**Figure 1H**). We confirmed the presence of the targeted allele in skin, liver, and tail of this mouse by PCR amplification of *lacZ* (**Figure 1I**). The phenotype in this mosaic male chimeric animal is consistent with the clinical presentation of FDH in males, who have somatic mosaic mutations [8], [9], [28]. The low survival of male chimeras is also in agreement with recent data showing that injection of CSD256 ES cells into tetraploid blastocysts results in early embryonic lethality and extensive gastrulation defects [27], [29]. This data provided the rationale for generation of a conditionally targeted *Porcn* allele to study *in vivo* effects of mammalian *Porcn* inactivation.

### Intronic targeting of a neomycin resistance minigene into the *Porcn* locus results in developmental defects in chimeric mice

**Figure 2:**
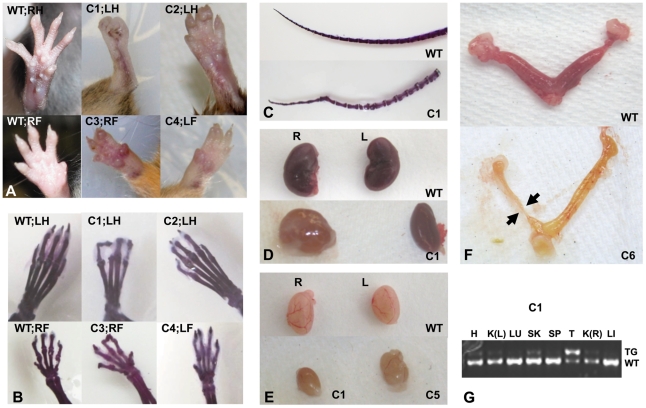
Phenotype of *Porcn-ex3-7-Neo-flox* chimeric mice. (**A**) Hypoplastic, fused, and missing digits on right (R) and left (L) fore- (F) and hindlimbs (H) in different chimeras (C1–C4) compared to wild type (WT). (**B**) Skeletal preparations of extremities shown in panel A. (**C**) Vertebral abnormalities in the tail of chimera 1 (C1). (**D**) Hydronephrosis of the right kidney and normal left kidney in C1. (**E**) Hypoplastic testicle in C1 and normal testicle in C5. (**F**) Uterine abnormalities: asymmetrical hypoplastic uterine horn in C6. (**G**) Gel picture showing amplification of the targeted allele in various tissues of C1. H, heart; K(L), left kidney; LU, lung; SK, skin; SP, spleen; T, testis; K(R), right kidney; LI, liver; TG, targeted allele; WT, wild type allele.

We next inserted an *FRT*-flanked *neomycin* (*Neo*) gene, a *loxP* site in intron 2, and a second *lox*P site in intron 7 of *Porcn* (**Figure S1**) and electroporated them into mouse ES cells to generate X*^Porcn-ex3-7Neo-flox^*/Y ES cell clones. We obtained 5/288 (1.7%) correctly targeted clones from which 3 independent lines of chimeric mice were generated after successful blastocyst injections with different clones. Of 17 chimeric mice, there were 9 high-level male and female chimeras from 2 different clones that displayed a mild phenotype reminiscent of human FDH (**Figure 2A–G**). These mice had absent, fused, and shortened digits on 1 to all 4 of their extremities (**Figure 2A and B**), vertebral anomalies of the tail (**Figure 2C**), hydronephrosis (**Figure 2D**), small testicles in males (**Figure 2E**), and uteri with rudimentary uterine horns in females (**Figure 2F**). We confirmed the presence of the targeted allele in various tissues by PCR amplification with primers P1 and P2 (**Figure 2G**). Therefore, intronic integration of the *Neo* gene and *loxP* sites created at least a hypomorphic *Porcn* allele. The phenotype observed in chimeric mice further confirmed that as in human FDH, mosaic expression of mutant *Porcn* is sufficient to generate developmental abnormalities. We excised the *Neo* gene by mating X*^Porcn-ex3-7Neo-flox^*/Y chimeras with mice expressing *Flp* recombinase to generate X*^Porcn-ex3-7flox^*/X and X*^Porcn-ex3-7flox^*/Y mice (**Figure S1D**), who had no observable phenotype and were fertile.

### Zygotic inactivation of *Porcn* by *EIIa-Cre* and *Hprt-Cre* causes early embryonic lethality

To generate *Porcn-ex3-7del* alleles (**Figure S1E**) in the zygote and earliest stages of embryonic development, X*^Porcn-ex3-7flox^*/X mice were bred to homozygous X/Y;*EIIa-Cre*/*EIIa-Cre* mice, which express Cre recombinase under control of the *EIIa* viral promoter [30]. From 13 litters, we obtained 38 female and 40 male offspring, but only 3 male and 3 female liveborn mice carried the *EIIa*-*Cre* transgene together with a *Porcn-ex3-7flox* allele (**Table 1**) and had low-level mosaicism for the deleted *Porcn-ex3-7del* allele (not shown). The recovery of these mosaic floxed/deleted survivors can be explained by mosaic expression of the *EIIa-Cre* transgene as previously described [31]. We then bred these survivors to wild type mice, but only obtained 2 heterozygous females carrying the *Porcn-ex3-7del* allele out of 133 offspring total. These mice were viable and showed diminished hair growth, which was also observed in their mosaic parents, but they had no other defects. We propose that skewed X-inactivation patterns may explain the fairly mild phenotype in these heterozygous offspring. Timed matings yielded 14 female and 4 male live embryos at E10.5, but only 5 out of 14 female embryos carried the *Porcn-ex3-7del* allele; we did not recover any X*^Porcn-ex3-7del^*/Y embryos or liveborn mice, indicating that lethality of embryos with this genotype occurs before E10.5. This is consistent with data that became available during the course of this work that support an essential role for *Porcn* in gastrulation [25], [27].

**Table 1:** Liveborn offspring from breeding of X*^Porcn-ex3-7flox^*/X mice to 3 different *Cre* strains.

Mating	X*^Porcn-ex3-7flox^*/X with X/Y;*EIIa-Cre/EIIa-Cre*	X*^Porcn-ex3-7flox^*/X with X/Y;*Prx-Cre/-*	X*^Porcn-ex3-7flox^*/X with X/Y;*Krt14-Cre/-*
Number of litters (average litter size)	13 (6)	6 (7.3)	7 (6.6)
Female∶male ratio	38∶40	19∶25	19∶27
X*^Porcn-ex3-7flox^*/Y;*Cre*	3	10	7
X*^Porcn-ex3-7flox^*/X;*Cre*	3	2	2
X/Y;*Cre*	37	5	6
X/X;*Cre*	35	4	5
X*^Porcn-ex3-7flox^*/Y	n/a	5	6
X*^Porcn-ex3-7flox^*/X	n/a	6	5
X/Y	n/a	5	8
X/X	n/a	7	7

Because of the observed mosaic inactivation of *Porcn* with the *EIIa*-*Cre* transgene [31] and to further examine the embryonic defects in heterozygous mutant females in this animal model for FDH, we next bred floxed mice to mice that express Cre recombinase under control of the endogenous X-linked *Hprt* promoter that drives expression of Cre in oocytes and zygotes. Ubiquitous non-mosaic deletion of floxed alleles can be obtained when conditionally targeted mice are bred with either female or male *Hprt-Cre* mice [32]. We reasoned that breeding floxed X*^Porcn-ex3-7flox^*/X females to X*^Hprt-cre^*/Y males would yield heterozygous deleted females and allow us to study the full phenotypic spectrum of *Porcn* mutations. It would also allow us to address whether germline heterozygous mutations in females are usually embryonic lethal, since it has been proposed that most surviving human females with FDH have either non-random X-inactivation or postzygotic mosaic mutations [9].

**Figure 3:**
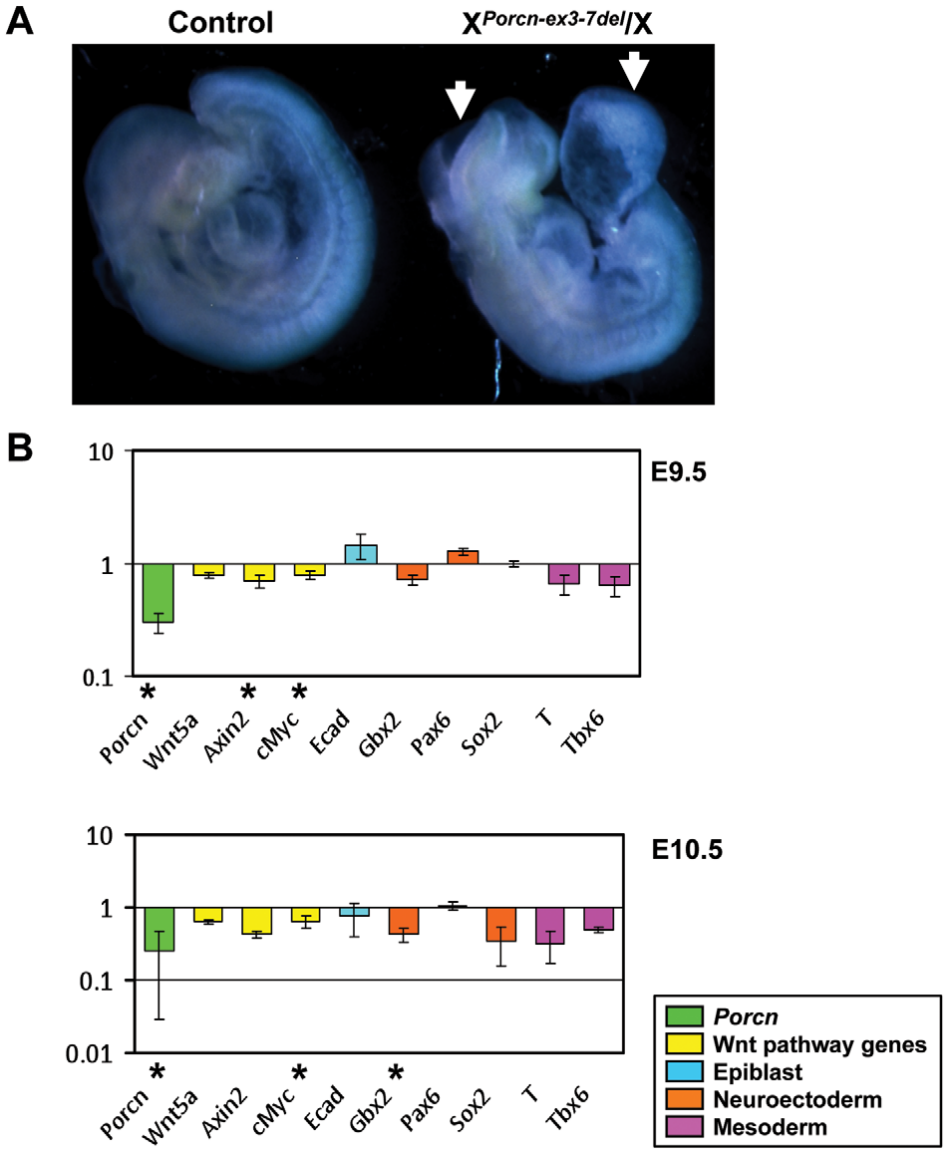
Phenotype at E9.5 and gene expression analysis of X*^Porcn-ex3-7del^*/X embryos. (**A**) X*^Porcn-ex3-7del^*/X embryo with open neural tube and abdominal wall closure defect (arrows). (**B**) Bar graph of quantitative real-time PCR analysis at E9.5 (top panel) and E10.5 (bottom panel) of *Porcn* wild type (*n* = 4 each) and mutant (*n* = 3 each) embryos. Bars are color-coded by gene type and indicate the log2 of the fold-deviation of gene expression levels in *Porcn* mutant embryos compared to wild type (set as 1). Error bars indicate standard errors of the mean. (*) indicates statistical significance at *p*<0.05 (Student's t-test).

From 5 litters, we obtained 17 liveborn male and 9 liveborn female offspring, none of which carried the *Porcn-ex3-7del* allele (**Table 2**), confirming that embryos with the X*^Porcn-ex3-7del^*/X genotype are lost prenatally. Timed matings revealed that 15 out of 43 live embryos at E9.5, 5 out of 18 at E10.5, and 3 out of 8 at E11.5 had the X*^Porcn-ex3-7del^*/X genotype. Most of these (>80%) had open neural tubes, defects in ventral body wall closure, and axial/tail truncation (**Figure 3A**). And mutant embryos appeared progressively smaller compared to wild type counterparts at advancing embryonic stages. By E12.5 most X*^Porcn-ex3-7del^*/X embryos were resorbed. These findings indicate that the majority of females with heterozygous inactivation of *Porcn* are lost prenatally, consistent with some clinical data in human FDH [9]. The axial/tail truncation and neural tube defect partially resemble Wnt3a mutants and the phenotype of conditional inactivation of β-catenin in 3 germ layers by Cre recombinase under control of a promoter fragment of the caudal-related homeobox gene (Cdx) [33], [34]. The severe ventral body wall defects in heterozygous female mice are very interesting in light of recent case reports of pentalogy of Cantrell in infants or fetuses with FDH and mutations in *PORCN* [35], [36]. The open ventral body wall was also reported in dermal β-catenin mutant mice and Dvl2;Dvl3 double mutant mice[37], [38].

**Table 2:** Liveborn offspring from breeding of X*^Porcn-ex3-7flox^*/X mice to *Hprt-Cre* mice.

Mating	X*^Porcn-ex3-7flox^*/X with X*^Hprt-Cre^*/Y
Number of litters (average litter size)	5 (5.2)
Female∶male ratio	9∶17
X*^Porcn-ex3-7flox^*/Y	9
X*^Porcn-ex3-7flox^*/X*^Hprt-Cre^*	0
X/X*^Hprt-Cre^*	9
X/Y	8

RNA from embryos collected at E9.5 and E10.5 was extracted to evaluate gene expression of *Porcn*, Wnt pathway, and Wnt target genes, as well as other developmental markers by quantitative RT-PCR. Expression of *Porcn* itself and the canonical Wnt signaling pathway gene *Axin2* and the target gene *c-myc* were significantly reduced at E9.5, indicating that reduced *Porcn* levels affects Wnt signaling and its targets (**Figure 3B**). Reduced expression of *Porcn* and *c-myc* persisted in E10.5 mutant embryos. There was also decreased expression of the neuroectoderm marker *Gbx2* (**Figure 3C**). *Gbx2* plays an essential role in cerebellum development and maintenance of the mid/hindbrain organizer and it is also required to pattern the neural tube [39], [40], [41]. The decreased levels of *Gbx2* suggest that reduced function of *Porcn* in X*^Porcn-ex3-7del^*/X embryos affects neuroectoderm development. This could in part explain the observed open neural tube defects. It also implies that, even though central nervous system anomalies and neural tube defects are not typically seen in human FDH patients, mutations of *Porcn* impair the development of these structures. We speculate that in humans these defects are too severe or associated with additional developmental defects to permit survival to birth.

### Conditional inactivation of *Porcn* in limb mesenchyme causes mesomelic limb shortening and digital abnormalities

**Figure 4:**
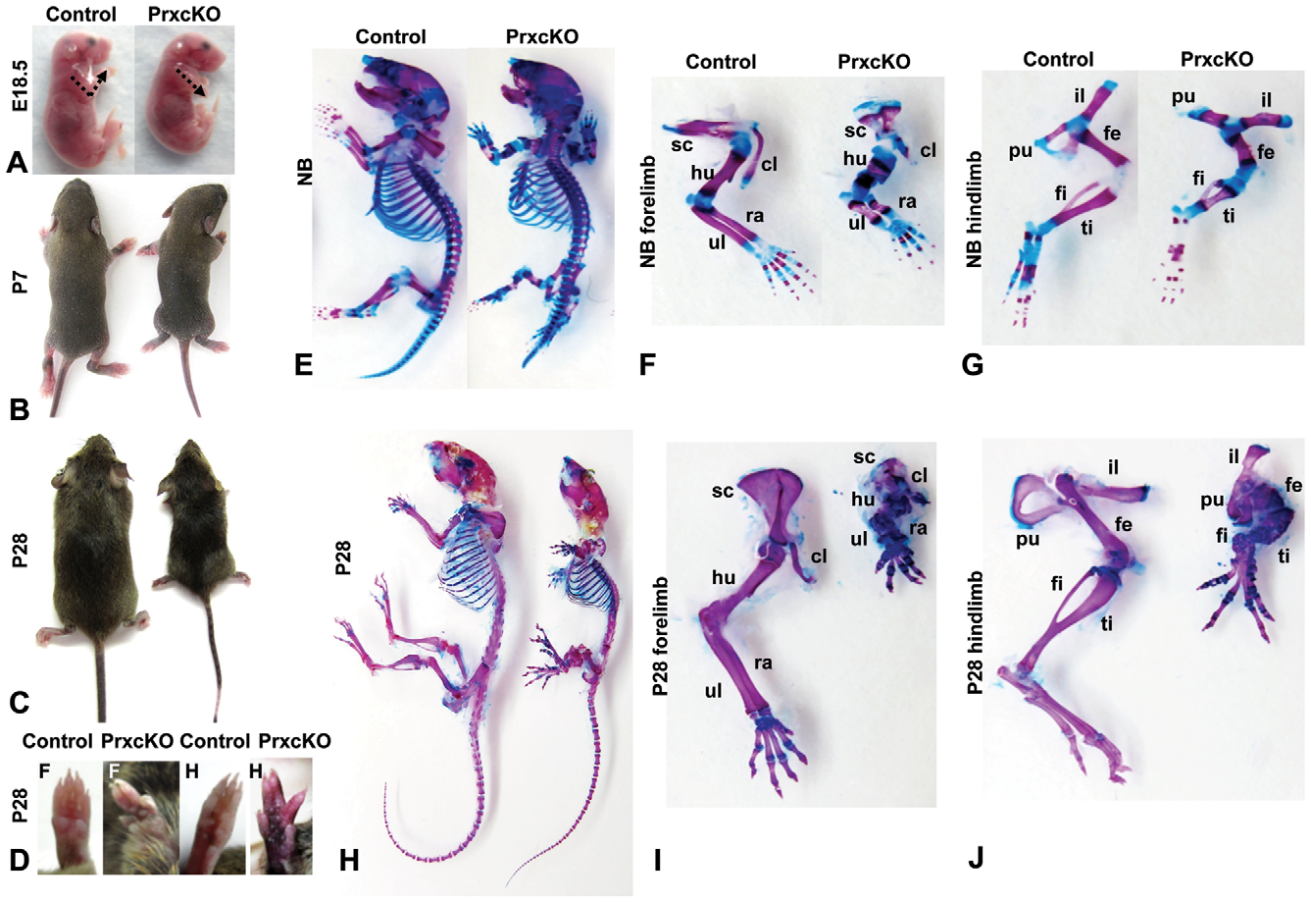
Phenotype of X*^Porcn-Ex3-7flox^*/Y;*Prx-Cre* mice. (**A**) Shortened forelimbs at E18.5 in X*^Porcn-Ex3-7flox^*/Y;*Prx-Cre* (PrxcKO) compared to wild type littermate (control). (**B**) Stunted growth at day 7 (P7) in PrxcKO compared to control littermate. (**C**) Appearance of PrxcKO compared to WT littermate at P28. (**D**) Digital abnormalities showing shortened limbs and shortened digits in PrxcKO compared to WT. F, forelimb; H, hindlimb. (**E**) Whole-skeleton preparations of newborn mice (NB). Mutant exhibits shortening of all skeletal elements. (**F, G**) Skeletal preparations of NB forelimbs and hindlimbs. (**H**) Whole-skeleton preparations of P28 mice. Mutant exhibits more dramatic shortening of all skeletal elements. (**I, J**) Skeletal preparations of forelimbs (F) and hindlimbs (H) at P28. hu, humerus; ra, radius; sc, scapula; ul, ulna; cl, clavicle; il, ilium; pu, pubis; fe, femur; ti, tibia; fi, fibula.

To further investigate the mechanisms by which loss of *Porcn* results in the limb defects of FDH, and to initiate studies into the role of *Porcn* in mesenchyme-derived tissues, we bred *Porcn-ex3-7flox* mice with *Prx-Cre* mice, reported to drive Cre expression in mesenchyme at early stages of forelimb development (**Figure 4**) [42]. Analysis of 6 litters (**Table 1**) indicated preserved Mendelian ratios in liveborn offspring. Male offspring (14/14, 100%) with X*^Porcn-ex3-7flox^*/Y;*Prx*-*Cre* genotypes and predicted *Porcn-ex3-7del* in tissues where *Prx-Cre* is expressed, have significantly shorter limbs as early as E18.5 (**Figure 4A**). They are also smaller than their littermates at postnatal day 7 (P7) (**Figure 4B**) and become increasingly stunted by P28 (**Figure 4C**). Their limbs remain very short with apparent syndactyly of digits of both fore- and hindlimbs in some animals (3/14, 21%) (**Figure 4D**). Skeletal preparations show that long bones of all extremities and digits are shortened, but we did not observe bony fusion of digits, indicating that the syndactyly is limited to soft tissues and does not include skeletal elements (**Figure 4E–J**). As expected, the *Porcn-ex3-7del* allele was detected by PCR in affected tissues from limbs of these mice (**Figure S2A**). These data indicate that *Porcn* deletion in mesenchymal derivatives is sufficient to cause severe defects of fore- and hindlimbs. As expected from the known expression pattern of *Prx-Cre* [42], defects were more severe in forelimbs. The shortened limb phenotype is similar to that of *Wnt5a* null except that our mice did not show loss of distal digits as Barrott et al. observed [25], [43]. This observation supports that *Porcn* may be required for “noncanonical” Wnt signaling; yet, the reason we observe a milder phenotype in X*^Porcn-ex3-7flox^*/Y;*Prx*-*Cre* mice remains to be discovered. The shortened limb phenotype models the features of severe cases of FDH, except for absence of bony syndactyly [44].

### Conditional inactivation of *Porcn* by *Krt14-Cre*-mediated deletion causes thinning of the skin with absent hair follicle development and dental defects

**Figure 5:**
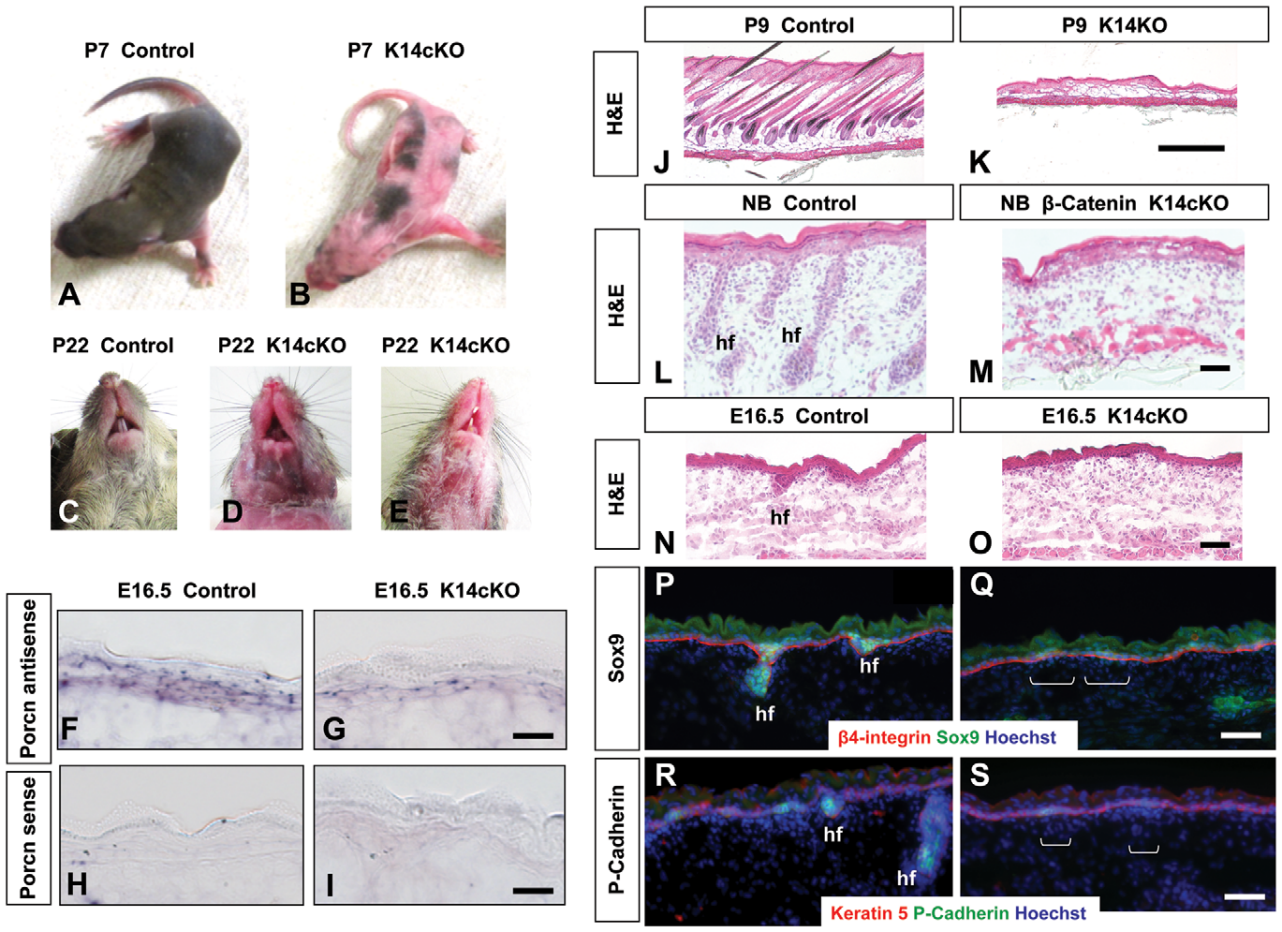
Phenotype of X*^Porcn-Ex3-7-flox^*/Y;*Krt14-Cre* mice. (**A, B**) Extensive alopecia of X*^Porcn-Ex3-7-flox^*/Y;*Krt14-Cre* (Krt14cKO) compared to wild type (control) at P7. (**C–E**) Variable dental phenotype (missing and hypoplastic teeth) in Krt14cKO mice. (**F–I**) RNA *in situ* hybridization with a *Porcn* antisense (F, G) and sense (H, I) riboprobe to E16.5 Krt14cKO (G, I) and control (F, H) skin showing reduced *Porcn* expression in mutant. (**J, K**) H&E staining of dorsal skin of Krt14cKO (K) and control (J) mice at P9 shows lack of hair follicles in the Krt14cKO mice. (**L, M**) H&E staining of dorsal skin from newborn (NB) *β-catenin* ^flox^/^+^;*Krt14-Cre* (β-catenin Krt14cKO; M) and control (L) mice also shows lack of hair follicles in the mutant. (**N, O**) H&E staining of skin from Krt14cKO (O) and control (N) mice at E16.5 shows that the early step of hair follicle morphogenesis does not take place in the Krt14cKO mice. hf, hair follicle. (**P, Q**) Immunostaining of skin at E16.5 shows that the Krt14cKO epidermis (Q) lacks Sox9-positive cells, which mark the hair follicle from the earliest budding stage (placode). (**R, S**) Skin of Krt14cKO epidermis (S) at E16.5 also lacks P-cadherin-positive cells, also indicating that placode formation is drastically compromised. Scale bars: F–I and L–S, 50 µm; J and K, 500 µm.

To identify the origin of defects of the skin and ectodermal appendages present in FDH and to investigate the contribution of altered ectodermal *PORCN* function to the FDH phenotype, we bred X*^Porcn-ex3-7flox^*/X mice with X/Y;*Krt14*-*Cre*/- mice. The *Krt14* promoter drives Cre expression in the basal layers of developing epithelia, causing deletion of floxed alleles in the epidermis and other ectodermal derivatives [45]. Mendelian ratios were preserved among liveborn offspring of these matings (**Table 1**). Heterozygous X*^Porcn-ex3-7flox^*/X;*Krt14*-*Cre*/- females had barely detectable hair loss, but males with *Krt14-Cre*-driven *Porcn* deletion had large areas of thin skin with alopecia (**Figure 5A and B**). The mosaic pattern of this phenotype likely results from variable expression of the *Krt14*-*Cre* transgene, as we have previously observed. Consistent with *Krt14*-driven Cre expression in multiple ectodermal derivatives, the mutant mice also had abnormally formed incisors (**Figure 5C–E**).

We detected the *Porcn-ex3-7del* allele in affected skin (**Figure S2B**) and confirmed reduced *Porcn* expression by RNA *in situ* hybridization with a *Porcn* riboprobe in the skin of E16.5 mutant embryos (**Figure 5F–I**). Histological analysis of skin at P9 showed that affected areas are completely devoid of hair follicles (**Figure 5J and K**). This phenotype is a phenocopy of that obtained when β-catenin is conditionally inactivated in skin by the same *Krt14-Cre* (**Figure 5L and M**), supporting the conclusion that it results from defective Wnt signaling. Analysis at E16.5 revealed that early budding of epidermal cells to form the hair placodes that should give rise to hair follicles did not take place in these embryos (**Figure 5N and O**). Absent staining in mutant skin for the early hair follicle marker P-cadherin and for Sox9, which is present in hair follicles from the placode stage, confirmed that placode formation was drastically compromised (**Figure 5P–S**), and indicates that hair follicle morphogenesis is blocked. This suggests that these aspects of the epidermal phenotype can result from ectodermal loss of *Porcn* (**Figure S2B**).

**Figure 6:**
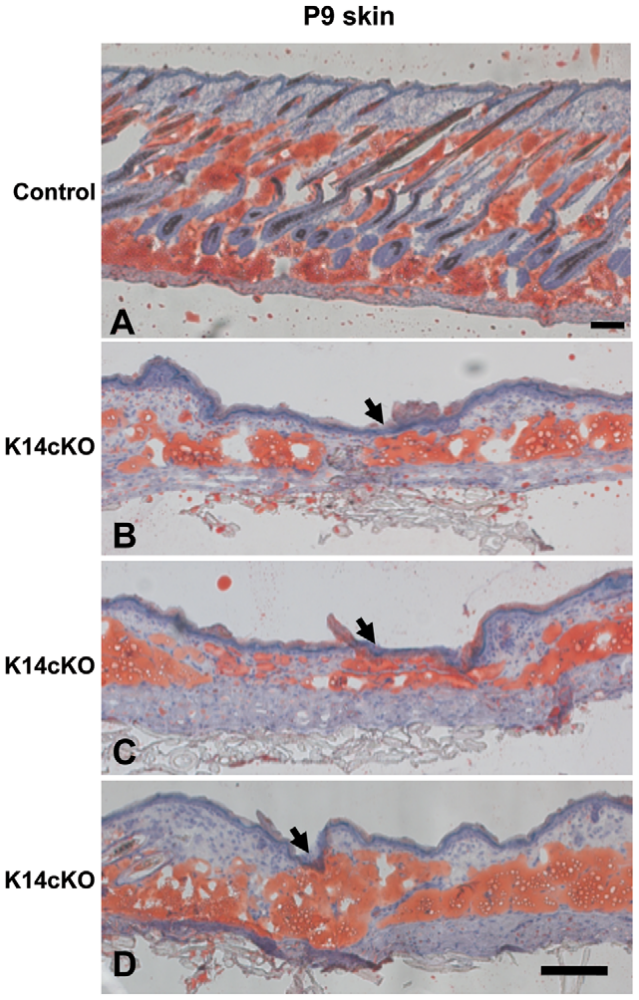
Oil red O staining of Krt14cKO skin at P9. (**A**) Control skin. Scale bar 100 µm. (**B–D**) Krt14cKO skin shows fat apposition close to the surface. Scale bars 50 µm.

Therefore, although *Prx* drives Cre expression primarily in mesenchymal derivatives and *Krt14* drives it primarily in ectodermal derivatives, mice with *Porcn* inactivation in either of these Cre lines have phenotypes that are remarkably similar to those seen in human patients, implicating that disruption of both mesenchymal and ectodermal *PORCN* contributes to the FDH phenotype. Interestingly, we also noted that in the areas of thinned skin, the subcutaneous fat was directly adjacent to the outermost epidermal layers of the skin (**Figure 6**). While this will require further detailed investigation, this finding may be relevant to the observed areas of apparent herniating fat found in the skin defects of patients with FDH.

### Effects of overexpressed wild type and mutant *PORCN* on cellular *WNT3A* secretion

**Figure 7:**
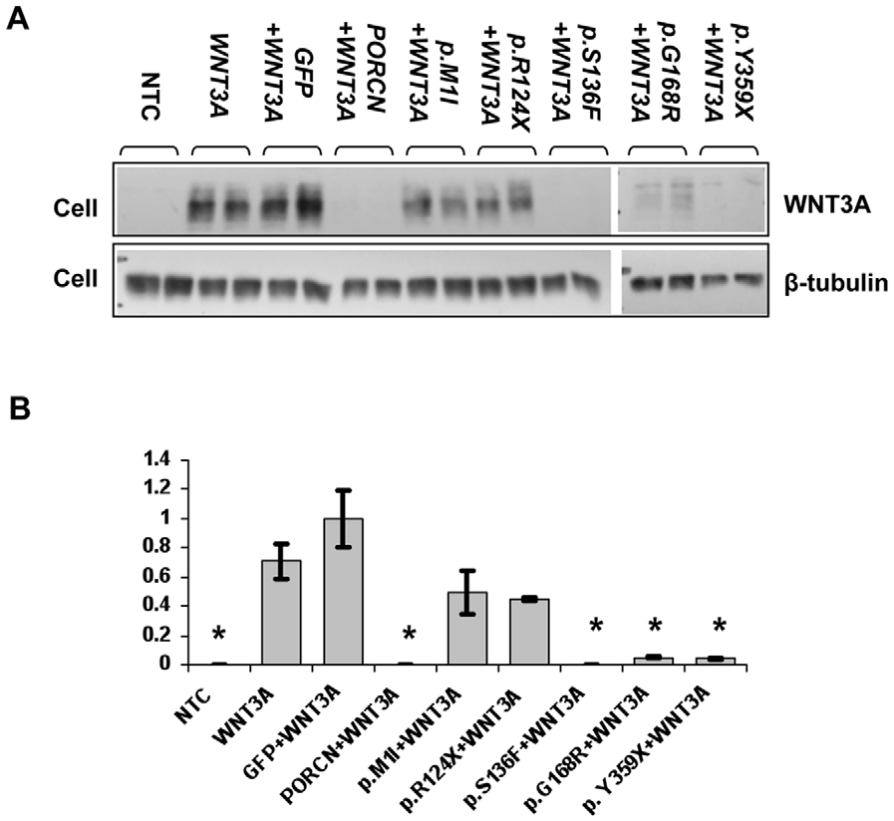
Cell-based WNT3A secretion assay. (**A**) Western blot showing WNT3A levels in total cell lysates after transient transfection. (NTC, non-transfected control). (**B**) Quantification of WNT3A levels in cell lysates. Co-expression of *WNT3A* with wild type *PORCN* (*WNT3A+PORCN*) results in disappearance of WNT3A from the cell lysate compared to *WNT3A* alone or *WNT3A+GFP*. In contrast, co-expression of mutant *PORCN* forms p.M1I and p.R124X with *WNT3A* causes WNT3A retention in cells, but other mutations, p.S136F, p.G168R, and p.Y359X, do not affect WNT3A secretion. We compared WNT3A levels in cells co-transfected by *WNT3A* and either wild type or mutant *PORCN* forms to WNT3A levels in cells expressing *WNT3A+GFP*, because these contain similar amounts of transfected DNA. Fold changes with standard deviation are shown; all data were normalized to β-tubulin; (*) indicates significant difference at *p*<0.05; the experiment was repeated 4 times.

It has been shown that *Drosophila* Porcupine and, in cell culture assays, its murine ortholog lipid-modify Wnt proteins (in particular Wnt3a) to promote their secretion from the ER and consequently from Wnt-producing cells, allowing them to act as signaling molecules that activate receptors on Wnt-responding cells [18], [22], [23]. However, a direct effect of Porcupine on WNT-protein secretion and accordingly a link between FDH-causing mutations and WNT signaling had not yet been demonstrated in human cells when we initiated our studies. We therefore performed a cell-based assay to investigate how human wild type (wt) and mutant *PORCN* influence secretion of WNT proteins (**Figure 7**). Co-expression of wt *PORCN* and *WNT3A* in human embryonic kidney (HEK293T) cells showed that, as expected, transiently overexpressed wt *PORCN* reduced WNT3A in the cell to virtually undetectable levels, indicating increased secretion of WNT3A. In contrast, *PORCN* with mutations p.M1I (c.3G>A) and p.R124X (c.370C>T), known to cause human FDH, resulted in WNT3A retention in cells compared to wt *PORCN* (**Figure 7A**). However, other *PORCN* mutations found in FDH, p.S136F (c.407C>T), p.G168R (c.502G>A), and p.Y359X (c.1077C>A), did not or only mildly affected WNT3A retention in cells. It remains to be investigated how these mutations affect WNT protein signaling. Interestingly, other recent studies also indicate that Porcupine proteins without lipid adducts are still secreted but have defective Wnt signaling [25], [46]. When the same assay was performed with overexpressed WNT1, which is also known to be modified by Porcupine, we found that disease-causing mutations of *PORCN* did not reduce WNT1 secretion as much as was seen with WNT3A (**Figure S3**). These results are in agreement with other non-human data demonstrating that the functional consequences of lipid modification of Wnt proteins by Porcupine varies between different Wnt ligands and that implications for Wnt signaling are complex and not limited to their secretion from the ER [19].

## Discussion

*PORCN* was first identified as the mutated gene in Goltz-Gorlin syndrome or FDH in 2007 [8], [9]. Since then, at least 80 different mutations and large genomic deletions of the *PORCN* gene have been found in FDH patients [36]. The resulting phenotype in human patients varies widely from mild skin and distal skeletal defects to severe forms of FDH, with multiple defects that include severe limb abnormalities involving long bones, aplasia cutis, limb-body wall complex anomaly, pentalogy of Cantrell, and Van Allen-Myhre syndrome [35], [36], [47]. Identification and characterization of *PORCN* mutations as the cause of FDH was important for genetic counseling and diagnostic testing in suspected cases. However, an animal model for the condition and functional cell-based assays are needed to determine the functional *in vivo* consequences of *Porcn* mutations on Wnt signaling and to investigate new therapies.

We first attempted to generate mice from ES cells with a genetrap insertion in the *Porcn* locus. This produced a single chimeric animal, but not unexpectedly, there was no germline transmission of this null allele. We then generated a conditional allele of Porcn by introducing *loxP* sites flanking exons 3 and 7. When targeted mice are bred with early embryonic Cre-expressing mice (*EIIa-Cre*, *Hprt-Cre*), ectoderm-specific *Krt14-Cre*, and limb mesenchyme-specific *Prx-Cre* mice, a variety of interesting phenotypes are observed that recapitulate various aspects of the human condition and prove that these mice provide a reliable animal model for human FDH. Our findings are in agreement with and significantly expand on those reported by Barrott and colleagues during the course of this work in a mouse model with a deletion of exons 2 and 3 of *Porcn* [25]. We also made important novel observations that may be relevant for other human conditions potentially resulting from *Porcn* mutations or other defects in the Wnt signaling pathway.

Although lethality of *Porcn* mutations in hemizygous males has already been confirmed in studies with embryos resulting from aggregation of CSD256 ES cells with tetraploid blastocysts [25] and *in vivo* [27], our data provides the first *in vivo* evidence that many heterozygous females with inactivating mutations of *PORCN* also die *in utero*. We also demonstrate that, even though neural tube defects are not typically present in liveborn humans with FDH, severe cranial neural tube defects are common in the heterozygous embryos. This is consistent with expression of Porcupine and Wnt proteins in the developing central nervous system and suggests that associated defects could be responsible for the lack of surviving humans with FDH that have NTDs or other severe brain abnormalities [34]. We also demonstrated in different ways that mosaicism for a *Porcn* mutation is important for the phenotype: we observed limb, skin, and internal organ abnormalities in the CSD256 genetrap chimera and in chimeric animals carrying the *Porcn-ex3-7Neo-flox* allele, as well as mosaic skin defects in mice with *Porcn-ex3-7* deletion driven by *Krt14-Cre* and by *EIIa-Cre*. Having the current mouse model will also allow us to study the contribution of X-inactivation patterns for the mosaic expression of the phenotype in females.

*Krt14-Cre*-mediated *Porcn* inactivation also causes complete absence of hair follicle development and differentiation. This is in agreement with the lack of hair follicles described with ubiquitous Cre recombinase-mediated inactivation [25], but our observation that inactivating *Porcn* only in ectoderm using *Krt14-Cre* is sufficient to cause this phenotype is novel. In addition, we found that the defects in hair follicle development phenocopy those observed after *Krt14-Cre*-driven β-catenin inactivation, implying that they are caused by defective canonical Wnt signaling in ectodermal derivatives. The absence of P-cadherin and Sox9 indicates a very early defect in specification of the hair placode. Our findings of abnormal dentition in the animals are also novel and may result from similar disruption in ectodermal Wnt signaling in the tooth placode.

Another novel observation is that the chimeric animals have some of the rarer phenotypic features occasionally described in FDH, such as urogenital anomalies. Considering the known role of Wnt proteins in the development of the urogenital tract, the *Porcn-ex3-7flox* mice will be valuable to study how Porcupine regulates Wnt signaling during the development of the urogenital tract.

Finally, the cell culture assays provide evidence that *PORCN* containing mutations that cause human FDH can influence the secretion and signaling of WNT proteins from the cell. Consistent with prior data [18], [25], [27], [46], we demonstrate that knockdown of *PORCN* by siRNA, as well as some of the FDH-causing mutations that we studied, cause retention of WNT3a in the cell. In contrast, similar experiments with WNT1 show a lesser and different effect on WNT protein secretion and indicate that the effects of identical mutations in *PORCN* cannot be generalized to all WNT proteins. A recent study also showed that differential palmit(e)oylation of two residues of Wnt1 has both overlapping and distinct consequences, whereby one is *Porcn*-dependent and the other is *Porcn* independent. It has been proposed that this differential residue modification may play a role in determining participation in the canonical versus non-canonical Wnt-signaling pathway [48]. This data provides further evidence that Porcupine has a complex role in the regulation of secretion and signaling of Wnt ligands and that individual Wnt-proteins are differently influenced. This is consistent with data by others that indicate that effects of Porcupine on Wnt signaling can be uncoupled from Wnt ligand secretion [19].

The mouse *Porcn* alleles that we generated, supported by cell-based assays, provide evidence for an important role of Porcupine in ectodermal and mesodermal signaling of Wnt proteins. This and other generated mouse models [25] will be important tools for further detailed characterization of the function of *PORCN* in development and disease, and have provided a model for investigating potential therapies for some of the features of FDH that progress postnatally, such as skin defects and peri-orificial papillomas.

## Materials and Methods

All animal procedures were approved by the Baylor College of Medicine Institutional Animal Care and Use Committee. All animals are monitored daily by experienced personnel, animal staff and veterinarians. Animals were euthanized when they were unable to stand or eat, or when they displayed agonal breathing, vocalization, self-mutilation, had dehiscent wounds, hypothermia, weight loss of >20%, or seemed otherwise to be suffering. For animals that were impaired in reaching standard food supply due to skeletal abnormalities, food pellets were placed on the bottom of the cage, and we confirmed that animals were reaching these pellets and were also able to reach drinking water.

### Generation and analysis of the *Porcn* genetrap mouse

Mouse embryonic stem (ES) cells (line CSD256) carrying a genetrap mutation in intron 2 of *Porcn* (**Figure 1A**) previously generated as described [49] were purchased from the Mutant Mouse Regional Resource Centers (MMRRC). ES cells were injected into blastocysts that were transferred into pseudopregnant females, yielding 1 male chimera out of 28 offspring. This mouse was sacrificed at 3.5 months of age and tissues were snap frozen for genetic analysis or fixed in 10% formalin and embedded in paraffin for sectioning and hematoxylin-eosin (H&E) staining for histopathology. Skeleton reconstruction of the bony skeleton (without cranium) was performed by micro-CT using a Gamma Medica FLEX X-O.X-SPECT SPECT/CT instrument (Gamma Medica Ideas Inc.) and analyzed with Amira 3.1.1 software. Genotyping of various tissues was performed by PCR-based detection of the *lacZ* gene present in the *β-geo* cassette of the inserted transgene.

### Construction of the targeting vector, embryonic stem cell (ES) culture, and gene targeting

The pFRT-LoxP plasmid used to construct the targeting vector was kindly provided by Dr. James Martin (Institute of Biosciences and Technology, Houston). The pFRT-LoxP contains 2 *loxP* sites, a neomycin gene flanked by two *FRT* sites, and a TK cassette for negative selection. The 4.7 kb 5′ targeting arm, 1.7 kb mid arm, and 5 kb 3′ targeting arm of the *Porcn* targeting vector and diagnostic probes for correct targeting were amplified from BAC clone bMQ-207H16 (129S7/AB2.2, Sanger Center) using TaKaRa *LA Taq*™ DNA Polymerase (Takara Bio, Inc.) and cloned into a TA-vector (TOPO® TA Kit, Invitrogen). A *Sca*I site for future characterization was introduced into the forward PCR primer used to amplify the 5′ arm. The 5′ arm was released from the TA-vector with *Pac*I and *Asc*I restriction digestion and cloned into *Pac*I/*Asc*I sites of the pFRT-LoxP plasmid; the mid arm was released with *Not*I and *Acc*65I, and digested and cloned into *Not*I/*Acc*65I sites of the pFRT-LoxP plasmid; the 3′ arm was released by *Xho*I restriction digestion and cloned into a *Xho*I site of the pFRT-LoxP plasmid to generate the *Porcn-ex3-7Neo-flox* targeting construct. This construct was sequenced to confirm the genomic DNA sequence and insertion sites, linearized by *Pac*I restriction digestion and electroporated into mouse ES cells of a 129S5/SvEvBrd genetic background. ES cells were cultured in medium with G418 (geneticin) and FIAU [1-(2-deoxy-2-fluoro-beta-D- arabinofuranosyl)-5-iodouracil] for positive and negative selection, respectively. DNA from surviving ES cell clones was isolated for restriction digestion with *EcoR*I and Southern analysis by hybridization with an external 3′ probe located downstream of the 3′ arm. Because of the presence of 5′ repeated sequences, the 5′ probe was located inside the 5′ targeting arm and was used after restriction digestion with *Sca*I for confirmation of results from the 3′ probe.

### Microinjection and mouse breeding

Three independent correctly targeted *Porcn-ex3-7Neo-flox* ES cell clones were injected into the cavities of C57BL/6J blastocysts to generate chimeras. Male chimeras were bred to C57BL/6J females to test for germline transmission of the targeted *Porcn-ex3-7Neo-flox* allele. They were also bred to *FLPeR* mice (129S4/SvJaeSor-*Gt(ROSA)26Sor^tm1(FLP1)Dym^*/J, obtained from Jackson Labs) to generate *Porcn-ex3-7flox* mice with the *Porcn* floxed allele. Heterozygous *Porcn-ex3-7flox* females were bred with various mice expressing Cre recombinase to generate *Porcn* conditional knockout mice. *Prx-Cre* (Tg(Prrx1-cre)1Cjt), *Hprt-Cre* (129S1/Sv-*Hprt^tm1(cre)Mnn^*/J) and *EIIa-Cre* (Tg(EIIa-cre)C5379Lmgd) were also obtained from Jackson Labs. The β–catenin floxed mice were obtained from Walter Birchmeier and the *Krt14-Cre* mice from Elaine Fuchs.

### Mouse genotype analysis by PCR

The positions of the genotyping primers are indicated in **Figure S1**. Primer sequences are P1: CAAGTCCTTCCGCATATGGT, P2: TGCATGCTTCAGGTAAGACG, P3: TACCGCACAGAGACCAAGTG and P4: CCACACTCAGGGGAGTCAGT. PCR reactions were performed on DNA extracted from tail-tip, skin, or other tissues using the following conditions: 1 cycle at 95°C for 3 minutes, 35 cycles at 95°C for 30 seconds, 58°C for 30 seconds, and 72°C for 40 seconds, and 1 cycle at 72°C for 5 minutes. Primer sequences for genotyping *EIIA-Cre*, *Prx-Cre*, and FLP mice were obtained from Jackson Labs. Primers for genotyping *Krt14-Cre* mice are CreF: TGCTGTTTCACTGGTTATGCGG and CreR: TTGCCCCTGTTTCACTATCC.

### Skeleton preparations, hematoxylin-eosin staining, Oil red O staining, RNA in situ hybridization and immunofluorescence

Skeletons were prepared following the standard Alcian Blue and Alizarin Red staining protocol. Hematoxylin-eosin and immunofluorescence staining were done on 4% PFA-fixed 6–10 µm sections of OCT-frozen dorsal skin or embryos. Oil red O staining was performed on Krt14cKO and control 4% PFA-fixed 6–10-µm sections of OCT-frozen dorsal P9 skin following standard protocol. Automated non-radioactive RNA *in situ* hybridization with an antisense *Porcn* riboprobe that is predicted to detect all known transcript variants and a sense control riboprobe was performed on skin at mouse embryos at E16.5 as described [27]. Primers for generating the riboprobe were prepared according to Biechele, et al. [27]. For immunofluorescence analysis, block-diluent solution of 5% normal donkey serum, 2% gelatin, and 0.2% Triton X-100 in PBS was used with primary antibodies to Sox9 (1∶100, Santa Cruz Biotechnology), b4-integrin (1∶200, BD Biosciences), P-cadherin (1∶200, R&D Systems), and Keratin 5 (1∶500, gift from Colin Jamora) and FITC- or RRX-conjugated donkey secondary antibodies (1∶150 and 1∶200, respectively, Jackson Labs). Nuclei were stained with Hoechst.

### *WNT3A* and *WNT1* secretion assay

Human *PORCN* isoform B (pDONR223-*PORCN*, ID:7828), *WNT3A* (pCR-BluntII-*WNT3A*, ID: 40007188) and *WNT1* (pCR4-*WNT1*, ID: 30915309) cDNA clones were purchased from Open Biosystems. All inserts were PCR-amplified and subcloned to pcDNA3.3-topoTA vector (Invitrogen). Since *PORCN* isoform D is the longest *PORCN* transcript, we used it to investigate the function of *PORCN*. Isoform D clones were generated by inserting an 18 bp fragment into the *PORCN* B sequence using the QuickChange® II Site-Directed Mutagenesis Kit (Agilent Technologies). *PORCN* isoform D mutants (p.M1I, p.R124X, p.S136F, p.G168R and p. Y359X) were also generated using this kit. Control plasmid pcDNA3.1-GFP was generously provided by Laura W. Burrus (San Francisco State University). HEK293T/17 cells (CRL-11268™, ATCC) were cultured in 6-well plates to 50% confluence. For each well, 500 ng of *WNT3A* or *WNT1* was co-transfected with 500 ng of wild type or mutant *PORCN* isoform D plasmids using the PolyJet™ DNA *In Vitro* Transfection Reagent (SignaGen). After 48 hours, cells were harvested and cell lysates were prepared for Western blot analysis. The assay was performed at least 4 times with 2 or 3 independent replicates for each. Rabbit polyclonal antibodies to Wnt3a (ab28472, Abcam), Wnt1 (ab15251, Abcam) and β-tubulin (ab6046, Abcam) were used for Western blotting. Quantification was done using ImageJ software (U.S. National Institutes of Health).

### Gene expression analysis

RNA from E9.5 and E10.5 *Hprt-Cre*-driven conditional knockout embryos was extracted with the miRNeasy Mini Kit (217004, Qiagen). Total RNA was reverse transcribed using the qScript cDNA Supermix (Quanta BioSciences, Inc.). RT-qPCR was performed with the PerfeCTa SYBR Green FastMix (Quanta BioSciences, Inc.) on the StepOnePlus Real-Time PCR System (Applied Biosystems), according to the manufacturer's instructions. Expression changes were quantified relative to 18sRNA as an endogenous control, using the 2^−ΔΔCT^ method. Primer sequences for *Porcn*, *Wnt5a*, *C-myc*, *Axin2*, *E-cadherin*, *Gbx2*, *T*, and *Tbx6* have been described previously [27].

## Supporting Information

Figure S1 **Generation of the** ***Porcn*** **targeted alleles.** (**A**) “Porcn locus” represents the wild-type (WT) locus; “Targeted Allele” contains *lox*P sites in introns 2 and 7 and an FRT-flanked *neomycin* (*Neo*) gene in intron 7 (*Porcn-ex3-7-Neo-flox*); “Floxed Allele” retains only *lox*P sites after excision of *Neo* (*Porcn-ex3-7flox*); “Deleted Allele” lacks exons 3 through 7 (*Porcn-ex3-7del*). P1-P4 indicate the lcoation of the various genotyping primers; blue boxes represent the probes for southern analysis; E = *Eco*RI and S = *Sca*I restriction sites; sizes for diagnostic fragments for southern analysis are also shown. (**B**) Southern analysis of ES-cell genomic DNA digested with *Eco*RI and hybridized with the 3′probe showing the 4.8-kb WT fragment, the 9.1-kb targeted (TG) *Porcn-ex3-7-Neo-flox* fragment, and the 7.2-kb *Porcn-ex3-7flox* fragment, obtained after transfection of correctly targeted X*^Porcn-ex3-7-Neo-flox^*/Y ES cells with a *Flpe*-expressing plasmid. (**C**) Amplification of the targeted *Porcn-ex3-7-Neo-flox* allele (439 bp) using primers P1 and P2. (**D**) Amplification of the *Porcn-ex3-7-Neo-flox* allele (407 bp) using primers P3 and P4. (**E**) Amplification of the *Porcn-ex3-7del* deleted allele (365 bp) using primers P1 and P4 (WT: wild type, TG: targeted, Flox: floxed, Del: deleted).
(TIF)

Figure S2 (**A**) Genotyping results by PCR of Prx-Cre conditional knockout mice. Amplification of the *Prx-Cre* (Cre), *Porcn-ex3-7-flox* (Flox), *Porcn-ex3-7del* (Del), and WT alleles in the *Prx-Cre* conditional knockout (*PrxcKO*) (M, 1 kb ladder; F, forelimb; H, hindlimb). (**B**) Genotyping results by PCR of K14-Cre conditional knockout mice.Amplification of the *K14-Cre* (Cre), *Porcn-ex3-7-flox* (Flox), and *Porcn-ex3-7-del* (Del) alleles in the *K14-Cre* conditional knockout (*K14cKO*) (M, 1 kb ladder; 1–3, skin samples from 3 mice).
(TIF)

Figure S3 **Cell-based WNT1 secretion assay.** (**A**) Western blot showing WNT1 levels in total cell lysates after transient transfection. Co-expression of WNT1 with wild type PORCN (WNT1+PORCN) results in disappearance of WNT1 from the cell lysate compared to WNT1+GFP (NTC, non-transfected control). Co-expression of mutant PORCN forms with WNT1 didn't cause significant WNT1 retention in cells compared to wild type PORCN. (**B**) Quantification of WNT1 levels in cell lysates. WNT1 levels in wild type and mutant *PORCN* co-transfected cells were compared to those in cells expressing WNT1+GFP because WNT1+GFP contains same amount of DNA as WNT1+PORCN or Mutant; all data were normalized to β-tubulin. Fold changes with standard deviation are shown. (* indicates significant difference at *p*<0.05; the experiment was repeated 4 times).
(TIF)
