# Supplementary figures and images for: Deletion of Porcn in Mice Leads to Multiple Developmental Defects and Models Human Focal Dermal Hypoplasia (Goltz Syndrome)
Source: PLoS One. 2012 Mar 6;7(3):e32331. doi: 10.1371/journal.pone.0032331 (PMC3295752; doi:10.1371/journal.pone.0032331)

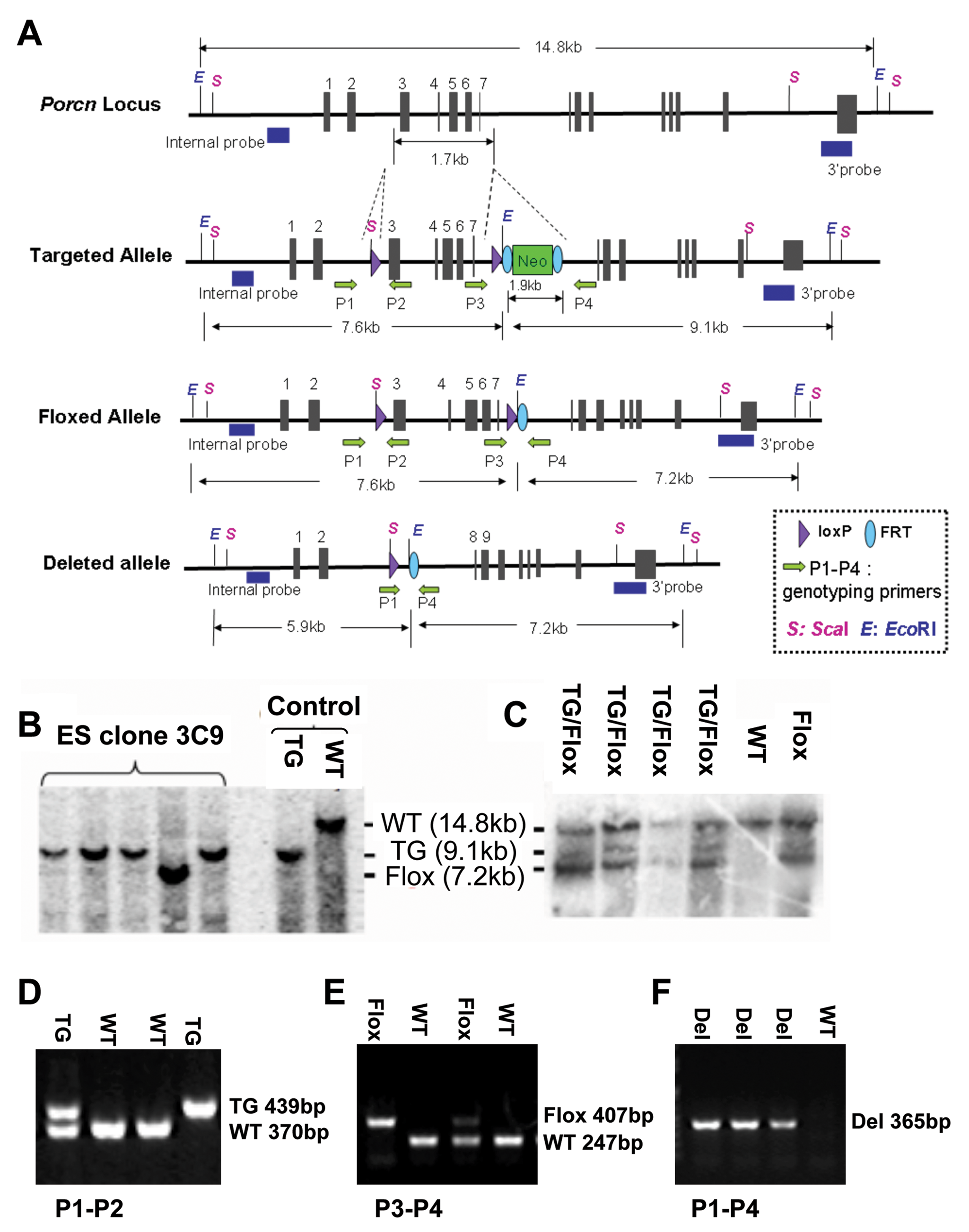

Supplement: Figure S1 — Generation of the Porcn targeted alleles. (A) “Porcn locus” represents the wild-type (WT) locus; “Targeted Allele” contains loxP sites in introns 2 and 7 and an FRT-flanked neomycin (Neo) gene in intron 7 (Porcn-ex3-7-Neo-flox); “Floxed Allele” retains only loxP sites after excision of Neo (Porcn-ex3-7flox); “Deleted Allele” lacks exons 3 through 7 (Porcn-ex3-7del). P1-P4 indicate the lcoation of the various genotyping primers; blue boxes represent the probes for southern analysis; E = EcoRI and S = ScaI restriction sites; sizes for diagnostic fragments for southern analysis are also shown. (B) Southern analysis of ES-cell genomic DNA digested with EcoRI and hybridized with the 3′probe showing the 4.8-kb WT fragment, the 9.1-kb targeted (TG) Porcn-ex3-7-Neo-flox fragment, and the 7.2-kb Porcn-ex3-7flox fragment, obtained after transfection of correctly targeted XPorcn-ex3-7-Neo-flox/Y ES cells with a Flpe-expressing plasmid. (C) Amplification of the targeted Porcn-ex3-7-Neo-flox allele (439 bp) using primers P1 and P2. (D) Amplification of the Porcn-ex3-7-Neo-flox allele (407 bp) using primers P3 and P4. (E) Amplification of the Porcn-ex3-7del deleted allele (365 bp) using primers P1 and P4 (WT: wild type, TG: targeted, Flox: floxed, Del: deleted). (TIF) [file pone.0032331.s001.tif]

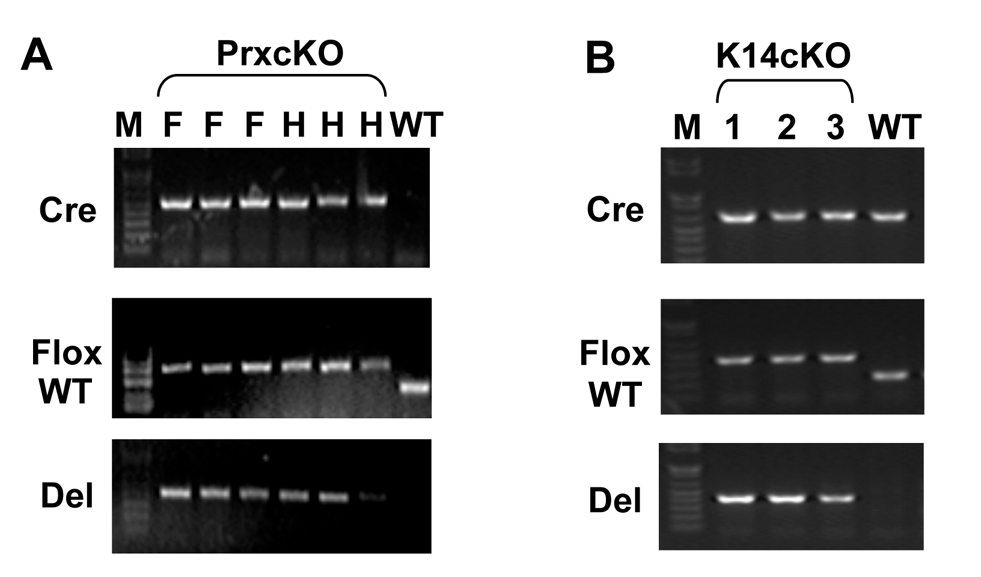

Supplement: Figure S2 — (A) Genotyping results by PCR of Prx-Cre conditional knockout mice. Amplification of the Prx-Cre (Cre), Porcn-ex3-7-flox (Flox), Porcn-ex3-7del (Del), and WT alleles in the Prx-Cre conditional knockout (PrxcKO) (M, 1 kb ladder; F, forelimb; H, hindlimb). (B) Genotyping results by PCR of K14-Cre conditional knockout mice.Amplification of the K14-Cre (Cre), Porcn-ex3-7-flox (Flox), and Porcn-ex3-7-del (Del) alleles in the K14-Cre conditional knockout (K14cKO) (M, 1 kb ladder; 1–3, skin samples from 3 mice). (TIF) [file pone.0032331.s002.tif]

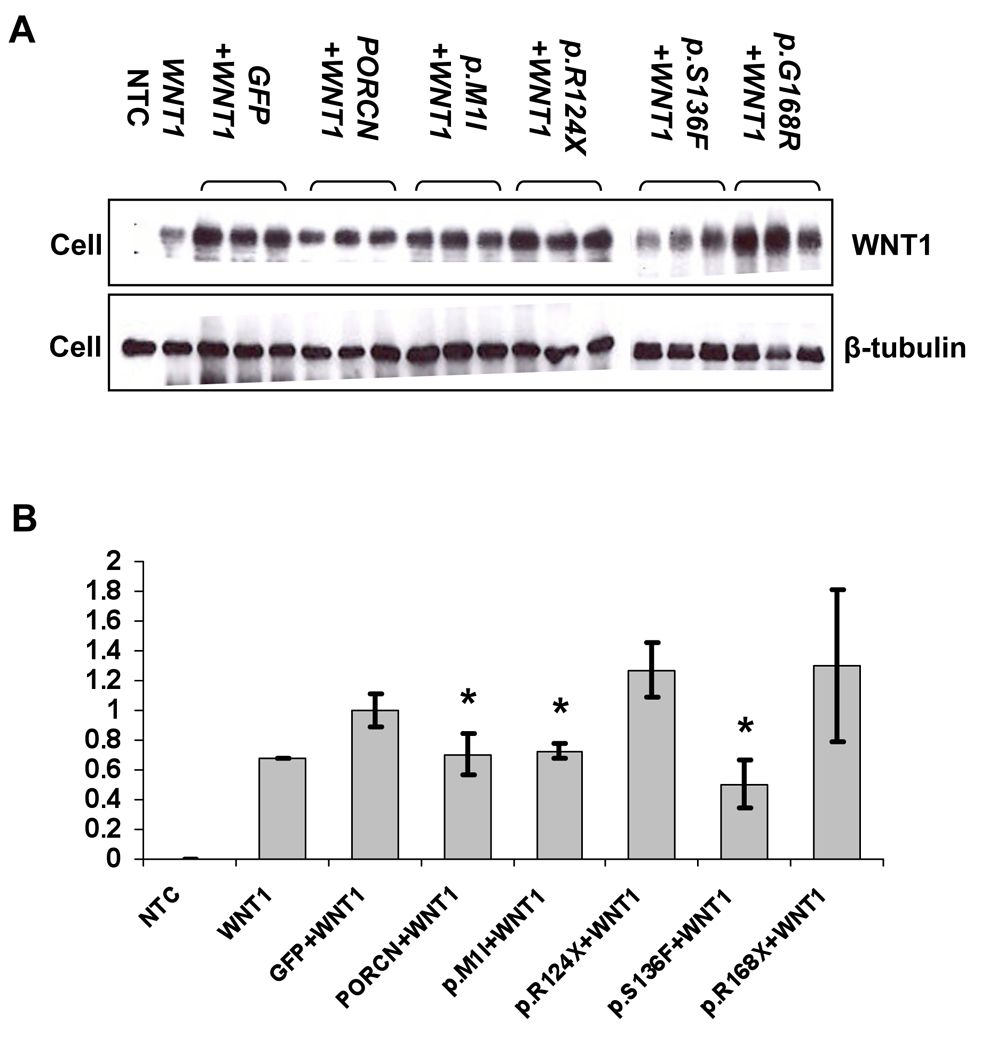

Supplement: Figure S3 — Cell-based WNT1 secretion assay. (A) Western blot showing WNT1 levels in total cell lysates after transient transfection. Co-expression of WNT1 with wild type PORCN (WNT1+PORCN) results in disappearance of WNT1 from the cell lysate compared to WNT1+GFP (NTC, non-transfected control). Co-expression of mutant PORCN forms with WNT1 didn't cause significant WNT1 retention in cells compared to wild type PORCN. (B) Quantification of WNT1 levels in cell lysates. WNT1 levels in wild type and mutant PORCN co-transfected cells were compared to those in cells expressing WNT1+GFP because WNT1+GFP contains same amount of DNA as WNT1+PORCN or Mutant; all data were normalized to β-tubulin. Fold changes with standard deviation are shown. (* indicates significant difference at p<0.05; the experiment was repeated 4 times). (TIF) [file pone.0032331.s003.tif]
